# Report from the 37th San Antonio Breast Cancer Symposium, 9–13th December 2014, Texas, USA

**DOI:** 10.3332/ecancer.2015.508

**Published:** 2015-02-04

**Authors:** M Ahmed, E Esposito

**Affiliations:** 1 Research Oncology, Division of Cancer Studies, King’s College London, Guy’s Hospital Campus, London SE1 9RT, UK; 2 Department of Clinical Medicine and Surgery, Breast Unit, University of Naples Federico II, Naples 80131, Italy

**Keywords:** San Antonio Breast Cancer Symposium 2014, endocrine therapy, hormone resistance, targeted therapy, genetics and genomics, supportive care, clinical chemotherapy, breast cancer screening, male breast cancer

## Abstract

The 37th San Antonio Breast Cancer Symposium (SABCS) was held at the Henry B. Gonzalez Conference Centre in San Antonio, Texas, USA between the 9 and 13th of December 2014. It brought together an interaction between basic scientists and clinicians involved in the management of breast cancer. The symposium included six general sessions, poster discussion, and poster sessions. The most important highlights in the fields of advancing endocrine therapy; hormone receptor positive advanced breast cancer and hormonal resistant therapy; targeted therapies; genetics and genomics; supportive (adjunct) care; chemotherapy treatments; breast screening and risk stratification; male breast cancer and future potential directions were included here.

## Original research presentations

### The latest updates on endocrine treatment of breast cancer

The conference provided significant advancement in the knowledge of endocrine treatment in terms of long-term outcomes, applicability to lobular cancer and the adjunct of ovarian suppression:

Dr Jack Cuzick from Queen Mary University, London, UK and colleagues presented the 16-year long-term follow-up of the IBIS-I breast cancer prevention trial of 7154 pre and postmenopausal women randomised to receive daily 20mg tamoxifen (*N* = 3579) or matching placebo (*N* = 3575) for five years [1]. They described how tamoxifen reduced the incidence of all breast cancer overall by 29% (hazard ratio (HR) = 0.71 (0.60–0.83), *P* < 0.0001). Invasive oestrogen receptor (ER)-positive (ER+) breast cancers were reduced by 35% (HR = 0.65 (0.53–0.80), *P* < 0.0001), but no effect was seen for invasive ER-negative (ER-) breast cancers (HR = 1.06 (0.71–1.58), *P* = 0.8). The overall risk reduction was similar in years 0–10 (HR = 0.71) and years 10–20 (HR = 0.70). No differences in breast cancer mortality were seen (24 tamoxifen versus 27 placebo; odds ratio (OR) = 0.89 (0.49–1.60), *P* = 0.7). All-cause mortality was non-significantly increased (173 versus 158, OR = 1.10 (0.88–1.38), *P* = 0.4), as were other cancers than breast (350 versus 315, OR = 1.12 (0.95–1.32); *P* = 0.2) in women randomised to tamoxifen.

Dr Michael Knauer from the Breast Cancer Centre, St Gallen, Switzerland and colleagues assessed the survival advantage of anastrozole compared to tamoxifen in lobular breast cancer in the ABCSG-8 study. They identified a significant improvement in overall survival (OS) for invasive lobular cancer treated with anastrozole compared to tamoxifen (HR 0.56, 95%CI 0.34–0.92) and this was replicated in disease-free survival (DFS) and OS for luminal B subtypes but not luminal A.

Dr Prudence Francis from the International Breast Cancer Study Group on behalf of colleagues described a randomised comparison of adjuvant tamoxifen (T) plus ovarian function suppression (OFS) versus tamoxifen in premenopausal women with hormone receptor-positive (HR+) early breast cancer (BC): analysis of the suppression of ovarian function (SOFT) trial [2]. A total of 3066 premenopausal women with HR+ BC were randomised to five years of tamoxifen versus tamoxifen+OFS. After a median follow-up of 67 months, five-year DFS was 86.6% in the T+OFS group and 84.7% in the tamoxifen group (HR = 0.83; 95% confidence interval (CI), 0.86–1.04; *P* = 0.10). In the chemotherapy cohort, five year overall survival was 94.5% for T+OFS and 90.9% for T (HR = 0.64; 95% CI, 0.42–0.96), with the cohort who remained premenopausal after chemotherapy experiencing a 4.5% absolute improvement in five-year breast cancer free interval (BCFI) with T+OFS versus T. This suggests that for the cohort of women who received chemotherapy and who remained premenopausal, the addition of ovarian suppression improved breast cancer outcomes. Dr Karin Ribi from the International Breast Cancer Study Group on behalf of colleagues then presented an assessment of patient recorded outcome measures of the SOFT trial and identified that overall patients receiving T+OFS experienced worse endocrine symptoms and sexual functioning than those receiving T alone during the first two years of treatment, but most differences between treatments were no longer apparent thereafter.

## Hormone receptor positive advanced breast cancer and resistance to hormonal therapy

The importance of the management of hormone receptor positive advanced breast cancer and hormone resistance was one of the conference’s priorities, with presentations addressing the latest advancements in this field.

Dr Ian Krop from the Dana-Farber Cancer Institute, Boston, MA on behalf of his colleagues commenced the FERGI phase II study of the PI3K inhibitor pictilisib (GDC-0941) plus fulvestrant versus fulvestrant plus placebo in 168 patients with ER positive, aromatase inhibitors(AI)-resistant advanced or metastatic breast cancer. They identified an overall progress-free survival (PFS) of 6.6 versus 5.1 months for the pictilisib and placebo groups respectively, although the study was only designed to be hypothesis generating, and hence it was not able to identify small differences between treatment arms. Dr Kerin Adelson from Yale University School of Medicine, New Haven, CT presented results of a randomised phase II trial of fulvestrant alone and then in combination with bortezomib (proteasome inhibitor) in ER positive metastatic breast cancer resistant to AI. Postmenopausal women with metastatic ER positive cancer resistant to AI were randomised either to fulvestrant alone or to the combination of fulvestrant and bortezomib, and a total of 118 women underwent randomisation. The PFS between the two arms significantly diverged after three months (*P* = 0.056). The addition of bortezomib to fulvestrant significantly reduced the rate of disease progression (HR 0.73, *P* = 0.06). Moreover the 12 months PFS rate improved from 14–28% (*P* = 0.03), whilst the six months PFS or the median PFS did not improve. The combination of fulvestrant to bortezomib was well tolerated with no grade 4 toxicity being observed. This trial provides proof for the principle that bortezomib may prevent or delay acquired resistance to endocrine therapy with fulvestrant.

Dr Rinath Jeselsohn from the Dana-Farber Cancer Institute, Boston, MA on behalf of her colleagues presented the TRANSCONFIRM phase III trial: A correlative analysis of advanced oestrogen receptor (ER) positive breast cancer. They demonstrated by subgroup analysis of the fulvestrant 500mg versus 250mg in endocrine patients of the CONFIRM trial [3] that high progesterone receptor (PR) level was associated with improved progression free survival (PFS), whilst positive HER-2 was associated with decreased PFS and also a trend existed for high Ki67 and decreased PFS. Surrogate predictors did not correlate with PFS or OS, high mRNA and protein levels of AP-2γ correlated with decreased response of fulvestrant and 37 gene signatures were able to cluster patients into good or poor responders to fulvestrant forming the basis for future work. Professor John Robertson from the University of Nottingham, UK and colleagues presented the overall survival data from the phase II FIRST Study on 500 mg fulvestrant versus anastrozole as first line endocrine monotherapy treatment for advanced breast cancer. A total of 205 postmenopausal ER positive patients were randomised either to 500 mg fulvestrant or to 1 mg anastrozole. The overall survival HR was 0.70 (95% CI 0.50–0.98, *P* = 0.041). Median OS for fulvestrant in this setting was 54.1 months versus 48.4 months for anastrozole (HR 0.70; 95% CI 0.50, 0.98; *P* = 0.041), demonstrating a survival advantage for fulvestrant over its competitors.

## Clinical trials of targeted therapies

The importance of targeted therapies to provide customised patient care —for HER-2 positive, triple negative and hormone receptor positive breast cancer— was demonstrated in a range of in-depth presentations including the application of targeted radiotherapy and additional treatments (PARP inhibition).

### HER-2+ disease:

Dr Teema Juntilla from the University of Washington discussed on behalf of his colleagues the potential application of HER-2 T cell dependent bispecific antibody (HER-2-TDB) for the treatment of HER-2 positive resistant breast cancer. The use of HER2-TDB can conditionally activate T-cells resulting in lysis of HER-2 expressing cancer cells and because of its unique mechanism of action unrelated to HER-2 signaling or sensitivity to chemotherapeutic agents, HER-2-TDB can potentially eradicate cells refractory to current HER-2 therapies. This warrants exploration within further pre-clinical models. Dr J Korkola from Oregon Health & Science University, Portland, OR and colleagues discussed the effect of sequential compared to concurrent treatment of pertuzumab plus T-DM1 (monoclonal antibody therapy) in HER-2+ breast cancer cell lines as a preclinical model system. They aimed to optimise scheduling and develop markers to predict response to pertuzumab and trastuzumab emtansine (T-DM1). They concluded that in pre-clinical testing cell lines, pre-treatment of pertuzumab diminished T-DM1 efficacy when compared to concurrent combination treatment, because pertuzumab then T-DM1 has been shown to be antagonistic in the great majority of the cell lines (17/21).

Dr Sara Hurvitz from the University of California, Los Angeles, CA and colleagues discussed the results of the BOLERO-1 trial, a phase III double blind, placebo-controlled multicentre trial of daily everolimus plus weekly trastuzumab and paclitaxel as first-line therapy in women with HER-2+ advanced breast cancer. The rationale for the BOLERO-1 trial is trastuzumab resistance in HER-2+ breast cancer is because of the hyperactivation of the PI3K/AKT/mTOR pathway. Between 2009 and 2011, 719 patients were randomised. The primary objective of PFS was not met. Median PFS prolonged by seven months in the ER negative subpopulation (20 months everolimus arm versus 13 months placebo arm, HR 0.66, *P* = 0.0049). However the protocol pre-specified analysis did not cross the statistical significance threshold. Higher rates of adverse events related deaths were reported for everolimus (3.6% versus 0% with placebo) and all but one occurred within 15 months of the study start. Dr Hurvitz concluded that data from BOLERO-1 validates the preliminary observation from BOLERO-3 [4] that treatment effect of everolimus differs based on ER expression in patients with HER-2+ advanced breast cancer in the absence of hormonal therapy.

Dr M Rimawi from Baylor College of Medicine, Houston, TX discussed on behalf of the Translational Breast Cancer Research Consortium (TBCRC) the results of the TBCRC023 trial, a randomised phase II neoadjuvant trial of lapatinib combined with trastuzumab, with or without endocrine therapy for 12 weeks versus 24 weeks in patients with HER-2 overexpressing breast cancer. No chemotherapy was given as this trial was based on targeted therapy only. The study was unable to reach the predefined primary endpoint to increase the pathologic complete response (pCR) to 45%. However the investigators demonstrated a twofold numeric increase in pCR in the 24 weeks arm over the 12 weeks arm. That increase was more than threefold in the ER positive subgroup, suggesting that longer treatment with dual anti HER-2 therapy in combination with endocrine therapy, and without chemotherapy, leads to a meaningful increase in pCR rate in ER+/HER-2+ breast cancer and warrants further study.

Dr Soonmyung Paik on behalf of the NSABP presented their assessment of intrinsic subtypes, PIK3CA mutation, and the degree of benefit from adjuvant trastuzumab in NSABP-31 [5]. However, they were unable to identify PIK3CA and PAM50 intrinsic subtypes as biomarkers for differential response to trastuzumab in NSABP B-31. This data suggest that results from the metastatic and neoadjuvant setting may not be always applicable to the adjuvant setting.

### Triple negative disease:

Dr Rita Nanda from the University of Chicago, IL described on behalf of colleagues the use of pembrolizumab (MK-3475) –a humanised IgG4, anti-PD-1 antibody- in advanced triple negative breast cancer. The PD-1 receptor-ligand pathway once activated can be used by tumours to evade immune surveillance, allowing neoplastic growth, therefore anti-PD-1 antibodies can potentially block such activation and reactivate the immune system to eradicate tumours. They demonstrated acceptable safety and tolerability profile in patients who were programmed death ligand 1 (PDL1) positive, advanced triple-negative breast cancer (TNBC) and an overall response rate (ORR) of 18.5% and median PFS of two months. A phase II study is planned to follow.

### Hormone receptor positive disease:

Professor Peter Schmid from the Barts Cancer Institute, London on behalf of colleagues presented the pre-operative window of opportunity study of pictilisb (GDC-0941) plus anastrazole versus anastrazole alone in 73 patients with ER positive, HER-2 negative operable breast cancer (OPPORTUNE study). They identified in their primary outcome measure a mean Ki67 suppression of 83.8% versus 66% for the pictilisib (plus anastrazole) and anastrazole only groups respectively (*P* = 0.004), suggesting the anti-proliferative benefit of dual compared to single agent treatment. Dr Kathy Albain from Loyola University, Chicago IL on behalf of colleagues described how the addition of gamma-secretase inhibitors (GSI) to endocrine therapy in a pre-surgical window study was able to produce significant biomarker responses, demonstrating that the GSI inhibited the Notch pathway and hence forming a therapeutic target for anti-Notch therapy in ER positive breast cancer.

### Radiotherapy targeting:

Dr Livi from Florence, Italy and colleagues presented the five year survival results of a phase III randomised trial comparing accelerated partial breast irradiation (APBI) using the intensity modulated radiotherapy (IMRT) technique versus standard whole breast irradiation (WBI). A total of 520 patients greater than 40 years of age who were diagnosed with early stage breast cancer were randomly assigned with a 1:1 ratio to either IMRT or to WBI arm. The experimental arm was treated with 30 Gy in five fractions (6 Gy/fr in two weeks), whilst the standard treatment consisted of 50 Gy plus a 10 Gy boost in 30 fractions (2 Gy/fr in six weeks). No statistical difference in terms of ipsilateral breast true recurrence (IBTR) rate was found between the two arms at five years. IBTR was 1.5% in the APBI arm compared to 1.4% in the WBI arm (long rank test *p* = 0.86). Five years overall survival was 99% in the APBI arm and 97% in the WBI arm. Moreover APBI group showed a statistically significant better safety considering any grade of skin toxicity (*P* = 0.0001) for acute and early late toxicity results and cosmetic results were shown to be better in the APBI arm (*P* = 0.045).

### Additional targeted therapy (poly ADP ribose polylmerase (PARP) inhibition):

Dr Yanghao Yu from Dallas, TX and colleagues discussed novel proteomic technologies focusing on ADP-ribosylation of PARP substrates [6]. They have developed the first large-scale approach to site-specific characterisation of the ADP-ribosylated proteome and identified 1,048 ADP-ribosylation sites on 340 proteins involved in a wide array of nuclear functions; among these were many previously unknown PARP downstream targets whose ADP-ribosylation was sensitive to PARP inhibitor treatment. They also confirmed that iniparib (the first putative PARP inhibitor to commence phase III clinical trials) had a negligible effect on PARP activity in intact cells. A deeper comprehension of the activity and the specificity of various PARP inhibitors at a preclinical level might allow breast cancer research to take a step forward for their introduction into the clinical practice. In terms of future directions this novel approach to site-specific characterisation of the ADP-ribosylated proteome is crucial and can serve as a predictive marker to predict sensitivity to PARP1 inhibitors.

## Genetics and genomics

The conference assessed the application of genetics and genomics for identification of therapeutic response and identification of potential drug targets.

Dr Obi Griffith from Washington University Medical School, St Louis, MO presented on behalf of his colleagues the prognostic effects of gene mutations in ER positive breast cancer from a cohort of 636 patients treated with tamoxifen monotherapy for five years and 10.4 years mean follow-up. They demonstrated significant associations between mutation status and improved survival for PIK3CA, ARID1B, ERBB3, MAP3K1, and GATA3 or worse survival for PTEN, DDR1, TP53, and JAK2. They suggested that associations with poor outcomes should be considered high priorities for pharmacological interventions. Pascal Gellert from the Institute of Cancer Research, London and colleagues assessed exome sequencing of post-menopausal ER-positive breast cancer treated pre-surgically with AIs in the perioperative endocrine therapy indvidualising care (POETIC) trial. In the treated group they identified that there were fewer mutations at surgery versus diagnosis (*p* < 0.026) and at the least one sub-clone was present in only one core-biopsy sample in 8/37 exome pairs suggesting spatial heterogeneity. Professor Michael Dixon from the University of Edinburgh, Scotland on behalf of colleagues presented their in-depth analysis of ER positive breast cancers during development of endocrine resistance and demonstrated ‘subtype shift’ from luminal B to A in progressor patients treated with neo-adjuvant AIs. They hypothesised that this may be reflective of reduced proliferative rates or a shift to the luminal A phenotype on AI therapy. Dr Ryan J Hartmaier from the University of Pittsburgh with colleagues performed a comprehensive analysis of genomic alterations in breast cancer progression (hormone-resistant recurrence). They identified a novel fusion gene between ESR1 (oestrogen receptor-α, ERα) and DAB2 (disabled-2) only in recurrent disease –confirmed on RT-PCR and western blot analysis– which they hypothesised conferred ligand independent cell growth and signaling. Further exploration of this genome wide binding of ESR1-DAB is yet to be performed.

Dr Giovanni Ciriello from Memorial Sloan-Kettering Cancer Centre, New York and colleagues performed a comprehensive molecular characterisation of invasive lobular breast tumours and developed a comprehensive atlas of genomic alterations revealing key recurrent mutational differences between invasive lobular breast cancer (FOXA1) from invasive ductal breast cancer (GATA3) tumourigenesis and a potential therapeutic target for invasive lobular breast cancer (Akt). These findings provide further insight into the molecular heterogeneity of ER positive breast cancer. Dr Christine Desmedt from the University of Brussels, Belgium and colleagues in their characterisation of lobular breast cancers discovered that CDH1, PIK3CA, TBX3, FOXA1, and the chromatin-related genes MLL2, MLL3, ARID1A, and ARID1B were more frequently mutated in ER positive/HER-2 negative invasive lobular cancer (*n* = 451) compared to the ER positive/HER-2 negative invasive ductal cancer (*n* = 266) from The Cancer Genome Atlas, whereas GATA3, TP53, and MAP3K1 were less frequently mutated. This provided an inisight into genomic alterations in lobular cancer and also avenues for targeted therapeutic responses.

Dr Christos Sotiriou from Brussels, Belgium on behalf of colleagues presented their assessment of the principles governing A-to-I RNA editing in breast cancer transcriptome. The exome and transcriptome of 58 breast cancer samples representing the four main known subtypes, namely TN, HER-2+, luminal A, and luminal B, and ten matched normal samples were sequenced using the Illumina platform. The editing frequency was found to be significantly higher in tumours compared to matched normal breast tissues demonstrating that A-to-I editing is a pervasive and well-controlled phenomenon in cancer that can drive aberrant transcriptome expression in breast and potentially the vast majority of cancers.

Dr Shailja Pathania from Dana-Farber Cancer Institute, Boston, MA and colleagues focused their research on replication stress suppression leading to a predisposition to hereditary breast cancer. They carried out a study on fibroblasts and mammary epithelial cells of BRCA1 mutation carriers and confirmed that a stalled fork repair defect was evident in the BRCA1 mutation carrier cells, meaning that *in vitro*, the inability to repair a stall fork in the BRCA1 carriers generates a kind of replication stress and genomic instability that eventually push the cells towards tumourigenesis.

## Trials of supportive care

The assessment of adjunct therapies to standard care to optimise patient outcomes was assessed.

Dr Brian Leyland-Jones on behalf of the EPO-ANE-3010 trial investigators discussed the results of the EPO-ANE-3010 trial, a non-inferiority phase III study aiming to demonstrate that erythropoietin plus standard supportive care is non-inferior to standard supportive care alone in anaemic patients with metastatic breast cancer receiving standard chemotherapy. The study aimed to rule out a HR of 1.15 for PFS (erythropoietin (EPO) versus control). It was recorded as 1.089 (95% CI 0.988–1.200) and did not meet protocol defined non-inferiority criteria of 1.15, showing a 9% increased risk. Time to progression (TTP) showed an observed 9% increased risk in disease progression (1.094 95% CI (0.991–1.209)). Thrombotic events were infrequent in both groups, but were doubled (2.8 versus 1.4) in the EPO group. It was concluded that for the management of the anaemia in the 1st and 2nd line chemotherapy treatment of metastatic breast cancer, transfusion would be the preferred approach. If the EPO is to be used that should be done very consciously, in limited circumstances, only after a careful assessment of risk and benefits.

Dr Chlebowsky from University of California, Los Angeles (UCLA), CA and colleagues presented the results of the final survival analysis from the Women’s Intervention Nutrition Study (WINS). They aimed to evaluate dietary fat reduction as adjuvant breast cancer therapy. A total of 2437 patients diagnosed with early stage breast cancer were randomly assigned 60:40 within one year after surgery to dietary intervention (target 15% calories from fat) or control from 1994 to 2001 with the primary endpoint of the study being relapse-free survival. The relapse-free survival HR for the dietary group was 0.76 (0.60–0.98) at 60 months, and the overall survival HR was 0.82 (0.64–1.07) at 108 months. WINS survival by ER positive group showed a HR of 1.1 (0.86–1.40 non statistically significant), whilst survival by ER negative group showed a HR of 0.60 (0.41–0.99 *P* = 0.045), and for ER and PR negative the HR was 0.46 (0.27–0.78 *P* = 0.006). This suggested favourable lifestyle influence on survival in hormone receptor negative subgroups as opposed to the full cohort.

## Chemotherapy directed trials

The latest developments in adjuvant chemotherapy trials and neo-adjuvant chemotherapy prognostic predictive studies were discussed.

### Adjuvant chemotherapy trials

Professor Michael Untch on behalf of colleagues from the German Breast Group presented late breaking abstract on a randomised phase III trial comparing nanoparticle-based (nab) paclitaxel with solvent-based paclitaxel as part of neoadjuvant chemotherapy in 1200 patients with early breast cancer. The team identified that whilst the side-effect profile of nab paclitaxel was significantly higher than solvent based, the primary endpoint of complete pCR was 38% versus 29% (*P* = 0.001) respectively. This warrants further follow-up to validate if this translates to better DFS and OS.

Professor Andrew Tutt from King’s College London, UK on behalf of colleagues discussed the treating of new targets (TNT) trial; a randomised phase III trial of carboplatin as compared with docetaxel for patients with metastatic or recurrent locally advanced TNBC. This trial randomised a total of 376 patients (188 to each arm). The researchers were unable to identify a significant difference between the two treatment groups using the primary endpoint (objective response rate), but were able to identify the subgroup of BRCA positive patients, who were significantly favouring carboplatin (*P* = 0.03).

Dr C Geyer on behalf of the National Surgical Adjuvant Breast and Bowel Project (NSABP) presented the NSABP-36 trial: A randomised phase III trial comparing six cycles of 5-fluorouracil (5-FU), epirubicin, and cyclophosphamide (FEC) to four cycles of Adriamycin and cyclophosphamide (AC) in patients with node negative breast cancer. They did not identify a significant difference between the two groups in terms of DFS, OS, recurrent-free interval (RFI) or distant recurrence-free interval (DRFI), however grade 3 and 4 toxicities were more frequently present within the FEC group.

Dr Joseph Sparano from the Montefiore Medical Centre, Albert Einstein College of Medicine, Bronx, NY on behalf of colleagues provided a ten year update on the E1199 trial: A phase II study of doxorubicin-cyclophosphamide followed by paclitaxel or docetaxel given every three week or weekly in patients with axillary node-positive or high risk node-negative breast cancer [7]. They were unable to identify a significant difference in DFS (*P* = 0.33) by taxane or schedule (*P* = 0.88), although there was a significant interaction between taxane and schedule (*P* = 0.007), favouring one-weekly paclitaxel (*P* = 0.011) and three weekly docetaxel (*P* = 0.001) to the standard arm of three-weekly paclitaxel in DFS and OS. When evaluated by subtypes in exploratory analyses, the one-weekly paclitaxel arm (but not the three-weekly docetaxel arm) was associated with improved DFS (*P* = 0.01) and OS (*P* = 0.019) in patients with TNBC.

Dr Gunter von Minckwitz on behalf of the German Breast Group presented the phase III ICE study: Adjuvant ibandronate with or without capecitabine in elderly patients with moderate or high-risk early breast cancer. They identified no difference in DFS at five years for the ibandronate + capecitabine versus ibandronate alone groups respectively (78% versus 75%, *P* = 0.7) and grade 3/4 toxicities of less than 2% with capecitabine except for hand-foot syndrome (6.8%).

### Prognostic indicators of adjuvant therapy

Dr Edith Perez from the Mayo Clinic, Jacksonville, Florida, presenting on behalf of her colleagues upon stromal tumour-infiltrating lymphocytes (S-TILS) in HER-2 positive breast cancer demonstrated that lymphocyte predominant (LPBC) HER-2 positive breast cancer when treated with chemotherapy alone was associated with a significant improvement in relapse-free survival (RFS) (*P* = 0.009) compared to non-LPBC but this was not replicated when chemotherapy and trastuzumab were combined (*P* = 0.79). Therefore, altering the percentage of S-TILS may prove beneficial in overall management as a future therapeutic option.

## Neo-adjuvant chemotherapy

The prognostic indicators of response to therapy including pathological complete response.

Dr Katherine Hoardley from the University of North Carolina and colleagues presented the mutational analysis of CALGB 40601 (Alliance), a neoadjuvant phase III trial of weekly paclitaxel (T) and trastuzumab (H) with or without lapatinib (L) for HER-2-positive breast cancer, in which they identified the presence of a TP53 mutation was significantly associated with achieving pCR (59% compared to 28% in wild type; OR = 3.7, *P* < 0.0001) which did not vary by treatment arm. Therefore, concluding that TP53 mutation is a frequent, clinically important event in HER-2-positive disease, and it predicts pCR to chemotherapy plus HER-2-targeting.

Dr Bruce Tromberg from the University of California, Irvine, CA on behalf of colleagues discussed the role of diffuse optical spectroscopic imaging (DOSI) in predicting pre-surgical neoadjuvant chemotherapy response in breast cancer. They used this to evaluate whether changes from baseline to mid-therapy in a DOSI-derived tissue optical index (TOI) could predict pCR in breast cancer neoadjuvant chemotherapy (NACT). Using -40% as a threshold for the DOSI-derived TOI they found that subjects in the group with a 40% or more decrease in tumour to normal TOI were more likely to be pCR (*P* = 0.0586, OR = 4.667, 95% CI: 0.945 to 23.038).

Dr William Sikov from Brown University, Providence, RI on behalf of colleagues presented their assessment of the impact of intrinsic subtype PAM50 and other gene signatures on pCR rates in TNBC after NACT +/- carboplatin (Cb) or bevacizumab (Bev) by performing PAM50 subtype analysis on 367 pre-treatment samples (of 443 patients who started NACT). The addition of Bev increased pCRs in basal-like tumours from 45–64% (*P* = 0.0009), while reducing pCRs in non-basal-likes from 60–43% (interaction *P* = 0.024); thus, a basal-like gene expression pattern was predictive of benefit from Bev. High expression of the HER-2 amplicon signature was uncommon and not prognostic for pCR overall but was associated with reduced benefit from Cb (interaction *P* = 0.025). High proliferation, high p53 mutation and low oestrogen signaling signatures were prognostic for higher pCR rates and predictive of the benefits from Bev (interaction *P* = 0.031, 0.0017, 0.0002, respectively).

Dr Justin Balko from the Vanderbilt University, Nashville, TN presented on behalf of colleagues their assessment of tumour-infiltrating lymphocytes (TILs) in post-treatment NACT triple negative tumours with residual disease. They identified a strong positive association of TILs in NACT-treated specimens. They observed it was with RFS (*P* = 0.0001) and OS (*P* = 0.0016). Genetic alterations in the Ras/MAPK pathway were associated with lower TILs in residual disease (*P* = 0.005) and suggestive of immune evasion and outcome in TNBC.

## New approaches to breast cancer screening and risk stratification of *in situ* disease

The use of additional ultrasound for breast screening and the stratification of patients with high risk in situ disease were discussed.

Dr Jein M Weigert from New Britain, CT presented on behalf of colleagues the retrospective Connecticut experiment of four years screening of women with dense breasts (mammographically >50% breast tissue) with additional ultrasound. She showed a stable incremental cancer detection of 3.2/1000 and an increased positive predictive value (PPV) rising from 7.1 in the first year to 17.2 by the fourth year. The cancer detection rate were continued at the same rate/1000 over the first four years. The improved PPV indicates that there is a learning curve in deciding which lesions to follow and which to biopsy. The uptake by eligible women remained steady at about 30%. Further to this long-term follow-up and cost assessment was planned.

Dr J Lipson from the Stanford University School of Medicine further discussed the role of supplemental screening for women with dense breasts, specifically focusing on ultrasound and tomosynthesis. She stated that the prevalence of women categorised as having dense breasts can be as high as 50% and this plays a major role in masking breast cancer during mammography. Notification law has been enacted in 19 states within the United States of America (USA) and insurance coverage is now mandated for supplemental screening tests in four states. Dr Lipson raised concerns upon the lack of long-term follow-up of additional screening measures to determine its true clinical impact —in terms of tumour stage at diagnosis and mortality– and the impact upon false positive rates from unnecessary interventions with additional ultrasound. She discussed how evidence was absent regarding cost-effectiveness of supplemental ultrasound. With specific regards to the tomosynthesis, Dr Lipson discussed that the addition of tomosynthesis to digital mammography is associated with a 15–30% decrease in false positive rate resulting in a reduction in recall rate and 30–50% increase in cancer detection, but long-term follow-up is required. Dr Lipson concluded that the benefits of additional screening measures are the increased cancer detection rate, whilst limitations of elevated false positive rates–much greater for ultrasound (four times greater than mammography) and much less for tomosynthesis.

Professor Eilen Rakovitch from the University of Toronto, ON presented on behalf of colleagues a population-based validation study of the ductal carcinoma *in situ* (DCIS) score predicting recurrence risk in individuals treated by breast conserving surgery (BCS). She described that traditional clinical and pathologic factors do not reliably identify individuals at low risk of recurrence after BCS. The investigators aimed to evaluate if the Oncotype DX DCIS score is associated with the risk of local recurrence (LR) in patients treated with BCS alone with negative margins in ER positive tumours and in all tumours regardless of ER status. The Ontario cohort (retrospective) of patients diagnosed with DCIS in Ontario between 1994 and 2003 was accessed and 571 tissue blocks reviewed. The DCIS score was associated with HR 2.26 (1.41–3.59, *P* < 0.001) in ER positive cancers and 2.15 (1.43–3.22, *P* < 0.001) in the full cohort. Multivariable analysis demonstrated age <50 years, tumour size >1 cm, solid subtype, and multifocality were found to be positively associated with the risk of LR. This demonstrated that DCIS score is associated with the risk of LR and invasive local recurrence in a population of patients with pure DCIS treated with BCS alone and may help in risk stratification of DCIS patients.

## Male breast cancer

Advancements in the characterisation and genomic mapping of male breast cancer were discussed.

Dr Fatima Cardoso from Champalimaud Cancer Centre, Lisbon, Portugal presented the first results of the EORTC 10085/TBCRC/BIG/ NABCG International Male Breast Cancer Programme. Cardoso and colleagues established a retrospective joint analysis of all male breast cancers diagnosed within the last 20 years and set up a prospective international registry running over 30 months from 278 breast centres. The data analysis showed that median age at diagnosis for males was 68.4 years and male breast cancer received more aggressive surgical intervention (96% of mastectomies and 76% of axillary clearance). Adjuvant radiotherapy was mostly correctly provided, but still 36% of N1 and 15% of N2 patients did not receive it. Male breast cancer presents usually as luminal A like subtype, 88% express androgen receptors, only 1% is triple negative, and 9% HER-2 positive. A total of 77% of male breast cancer is treated by adjuvant endocrine therapy—mainly tamoxifen (88%)– and the ER and PR status being prognostic with high expression associated with better outcomes.

Professor Jorge Reis Filho from Memorial Sloan Kettering Cancer Centre, New York, NY and colleagues discussed the genomic landscape of male breast cancers. They aimed to define a repertoire of somatic mutations in male breast cancers, using a platform containing the genes most frequently mutated in female breast cancer and genes involved in DNA repair. On the gene analysis of 59 patients diagnosed with male breast cancer, they found that the ER positive subgroup is characterised by somatic mutations in PI3K, GATA3, TP53 and MAP3K1, and that luminal B like tumours more frequently display GATA3 somatic mutations, mutations affecting DNA related-genes and 11q losses compared to luminal A cancers. From a comparative analysis of male breast cancer and luminal female breast cancer, it has emerged that PI3K and TP53 mutations are less frequent, but have similar patterns of gene copy alterations. Reis-Filho and colleagues concluded that genes mutated in male breast cancer are enriched for DNA repair-related genes and hypothesised that these mutations may in part account for the lack or the lower levels of TP53 mutations.

## Potential future directions

The novel and potential advancements in patient care, which maybe able to improve patient outcomes in the future are highlighted form the conference.

Professor Mary-Claire King from the University of Washington, Seattle, WA gave a thought-provoking lecture on the genomic analysis of inherited breast and ovarian cancer calling for a population-based screening approach to BRCA1 and BRCA2 mutations in women over 30 years of age as part of a routine medical care. The rationale was based on the evidence that 50% of patients who carry BRCA1 and BRCA2 mutations have no close family history of breast or ovarian cancer. Concluding that every breast and ovarian patient with a BRCA1 and BRCA2 mutation detected after diagnosis is a missed opportunity to prevent a cancer, Professor King declared that based on a UK cost-effectiveness analysis of population-based screening, costs would be absolutely acceptable, considering that market forces are driving down test expenses.

Professor Jorge Reis-Filho from Memorial Sloan-Kettering Cancer Centre, New York, NY discussed the highlights in translational research from 2014 focusing onto three main topics: genetics and genomics, intra-tumoural heterogeneity, and TILs. With regards to genetics, PALB2 (Partner And Localizer of BRCA2) was presented as a new high-risk breast cancer gene identified and the reasons why it should be added to genetic testing for BRCA1/BRCA2 were clearly explained. Breast cancer intra-tumoural heterogeneity was confirmed as a common phenomenon and a gradual process, especially in the metastatic setting. Finally, level I evidence for stromal TILs as a prognostic indicator for chemotherapy treated TNBCs has been shown, whilst in HER-2+ disease this concept remains to be fully defined. Professor Reis-Filho concluded that heterogeneity is a tractable challenge with novel technologies, and pointed out the need of immunotherapy biomarker development.

The increasing burden of clinically occult (non-palpable) breast cancers with extended screening programmes and advanced imaging modalities has meant that the development of novel alternative localisation techniques free from wire and radioisotope dependence has become more pertinent for outcome measures and patient-satisfaction within breast conservation. Dr Muneer Ahmed from King’s College London, UK discussed the use of a handheld magnetometer and magnetic tracer for the performance of lesion localisation and concurrent sentinel lymph node biopsy (SLNB) in the MagSNOLL trial in a small cohort of 20 non-palpable cancers. The initial promising results are to be further explored in larger numbers and an assessment of the longevity of the tracer *in vivo* could lead to additional benefit as a biopsy marker, avoiding multiple radiology attendances prior to surgery.
